# RNAi-Mediated Knockdown of IKK1 in Transgenic Mice Using a Transgenic Construct Containing the Human H1 Promoter

**DOI:** 10.1155/2014/193803

**Published:** 2014-01-12

**Authors:** Rodolfo Moreno-Maldonado, Rodolfo Murillas, Manuel Navarro, Angustias Page, Cristian Suarez-Cabrera, Josefa P. Alameda, Ana Bravo, M. Llanos Casanova, Angel Ramirez

**Affiliations:** ^1^Molecular Oncology Unit, Centro de Investigaciones Energéticas, Medioambientales y Tecnológicas (CIEMAT), 28040 Madrid, Spain; ^2^Department of Epithelial Biomedicine, CIEMAT, 28040 Madrid, Spain; ^3^Department of Veterinary Clinical Sciences, Laboratory of Pathology Phenotyping of Genetically Engineered Mice, Faculty of Veterinary Medicine, University of Santiago de Compostela, 27002 Lugo, Spain

## Abstract

Inhibition of gene expression through siRNAs is a tool increasingly used for the study of gene function in model systems, including transgenic mice. To achieve perdurable effects, the stable expression of siRNAs by an integrated transgenic construct is necessary. For transgenic siRNA expression, promoters transcribed by either RNApol II or III (such as U6 or H1 promoters) can be used. Relatively large amounts of small RNAs synthesis are achieved when using RNApol III promoters, which can be advantageous in knockdown experiments. To study the feasibility of H1 promoter-driven RNAi-expressing constructs for protein knockdown in transgenic mice, we chose IKK1 as the target gene. Our results indicate that constructs containing the H1 promoter are sensitive to the presence of prokaryotic sequences and to transgene position effects, similar to RNApol II promoters-driven constructs. We observed variable expression levels of transgenic siRNA among different tissues and animals and a reduction of up to 80% in IKK1 expression. Furthermore, IKK1 knockdown led to hair follicle alterations. In summary, we show that constructs directed by the H1 promoter can be used for knockdown of genes of interest in different organs and for the generation of animal models complementary to knockout and overexpression models.

## 1. Introduction

RNA interference (RNAi) is an evolutionarily conserved mechanism of regulation of gene expression in eukaryotic cells. It is based on the degradation of mRNAs by the action of complementary small interfering RNAs (siRNAs) and the concomitant decrease in the synthesis of the corresponding protein. siRNAs, approximately 21-nucleotide long, are produced in the cell from longer double-stranded RNAs or can be synthesised from small hairpin RNAs (shRNAs) transcribed from genetic constructs produced in the laboratory and introduced into the cells. RNAi has been used for the functional knockdown of specific proteins in several experimental systems, from cultured cells [1] to complete organisms, including mammals [2]. Typically, knockdown transgenic animals are produced by the introduction of the RNAi construct in the cells by lentiviral vectors or by pronuclear microinjection of one-cell embryos. It is also possible to generate knockdown animals from genetically modified ES cells [3]; for a review about the application of this technology to living mammals, see [2].

RNAi technology represents an interesting tool in functional genetic studies for several reasons. Firstly, it is potentially applicable for decreasing the expression of the protein of interest in species for which ES cells are not available. Secondly, RNAi usually results in partial inhibition of the expression (typically, in the range of 60–90%) and thus allows the generation of graded hypomorphic phenotypes, which are usually impossible to generate by ES gene targeting technologies [3, 4]. These hypomorphic animal models are complementary, and sometimes more informative, to loss-of-function knockout models, especially when gene inactivation causes embryonic lethality. In addition, both inducible and reversible expressions of siRNA can be achieved by using the same approaches used in other transgenic systems, such as Cre recombinase-controlled transgene expression and tetracycline responsive elements [5–8]. Finally, RNAi is a promising therapeutic tool that could be used for the silencing of dominant mutant alleles [9, 10], for silencing of mutant transcripts originated from alleles carrying triplet expansion in the 3′-untranslated region [11], or for the treatment of solid tumors in patients [12].

Different types of RNA polymerases (RNApol) are responsible for the production of different types of RNAs in eukaryotic cells. While RNApol II transcribes mRNA-coding genes, some microRNAs and other noncoding RNAs, RNApol III synthesizes, among others, transfer RNAs, 5S ribosomal RNA, and other short noncoding RNAs required in all cell types, such as U6 RNA—a component of spliceosomes—and H1 RNA—the RNA component of the RNase P ribonuclease, implicated in tRNA maturation. Thus, RNApol III products are involved in primary cellular processes such as transcription, RNA processing, and protein synthesis [13, 14]. For experiments involving siRNA synthesis, both RNApol II- and RNApol III-dependent promoters have been used. Constructs containing promoters transcribed by RNApol II are used when a tight regulation of transgene expression is desired (i.e., tissue-specific or inducible expression) [7, 15]. In general, as RNApol III promoters are shorter than RNApol II promoters, they are more suitable for constructs with length limitations, like those carried by viral vectors. Among the RNApol III promoters, mouse U6 and human H1 gene promoters are the most commonly used in transgenic constructs. Considering the expression pattern of these genes, it is generally accepted that their promoters drive ubiquitous and high-level expression of transgenes. In this regard, it is interesting to note that although there are several published reports describing the specific knockdown of the gene of interest using RNApol III promoters, the expression pattern of these transgenes has not been studied in detail in most of the studies. Other aspects of RNApol III-containing transgenes that have proven important in classic transgenic constructs containing RNApol II promoters, including sensitivity to integration position effects and the inhibitory influence of prokaryotic sequences [16], also remain poorly analysed.

The IKK1 protein is a key regulator of the NF*κ*B signalling pathway that plays a crucial role in epithelial physiology. IKK1 gene inactivation in mice leads to lack of keratinocyte differentiation, to defective function of the skin and other stratified epithelia, and to newborn lethality [17, 18]. Mice with IKK1 inactivation restricted to keratinocytes also die at birth [19]. These phenotypes preclude the study of IKK1 function in adult mice by using knockout models. The study of other animal models (i.e., IKK1+/− mice and transgenic mice overexpressing IKK1) has shed some light into additional functions of IKK1, indicating that IKK1 is important for the development of epidermal cancer [20–22].

To gain insight into IKK1 function in adult skin and to determine the feasibility of achieving a partial loss of function by RNAi expression in skin and other organs of transgenic mice, we microinjected a H1 promoter-driven RNAi construct directed against IKK1 into one-cell embryos. We detected in these transgenic mice variable expression levels of IKK1 siRNA in several tissues of different transgenic lines, in contrast to the roughly homogeneous activity of endogenous H1 promoter, suggesting that the expression of this type of transgenic constructs is subject to position effects. RT-qPCR analysis of IKK1 expression indicated a variable decrease in mRNA levels, ranging from 0 to 80%. In addition, some transgenic mice showed alterations in hair growth and in hair follicle morphology after depilation. Taken together, our results indicate the feasibility of RNApol III-transcribed genetic constructs for knockdown of specific proteins in different tissues of transgenic mice, although with variable efficiency when comparing different tissues or different animals.

## 2. Materials and Methods

### 2.1. IKK1 Interfering Plasmids

**Figure 1:**
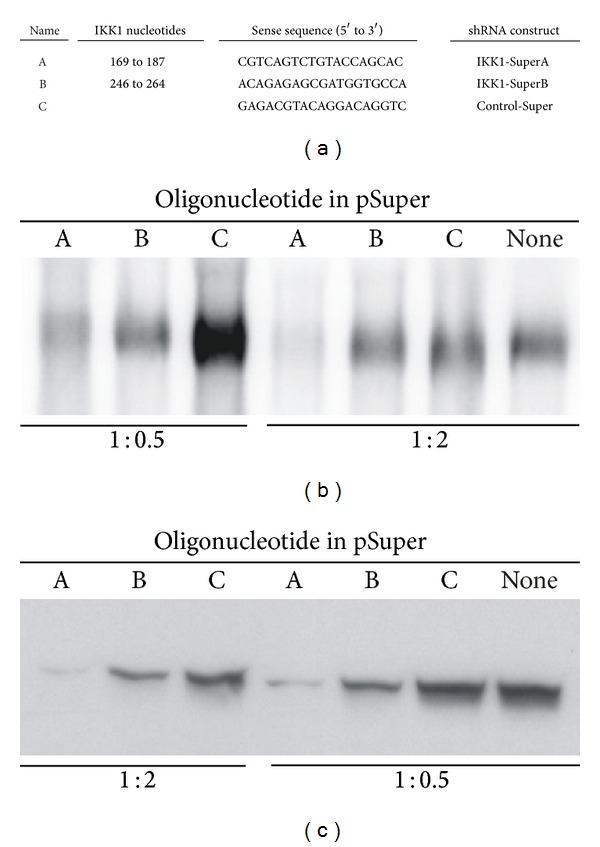
IKK1 silencing in HEK293T transiently transfected cells. (a) Sequences included in pSuper plasmid vector for the production of shRNAs designed to knockdown IKK1 expression. Two IKK1 specific oligonucleotides (A and B) and one control scrambled sequence (C) were cloned in pSuper and tested in transfected cells. Nucleotide numbers refer to RefSeq: MN_007700.2. (b) Northern blot analysis of transiently transfected HEK293T cells hybridized with a probe specific for IKK1. Plates were cotransfected with a plasmid for the expression of IKK1 and the indicated pSuper-derivatives; “none” indicates that an empty plasmid was used. (c) Western blot analysis of IKK1 produced in transfected cells. In (b) and (c), each lane contains pooled RNAs or proteins extracts from two different plates. Ratios between the IKK1 expression plasmid and the pSuper-derived interfering plasmid used in each transfection are indicated below the blots in (b) and (c).

For IKK1 interference, two different 64 bp long synthetic oligonucleotides containing IKK1-specific sequences were subcloned between the BglII and HindIII sites of pSuper vector (Oligoengine; Seattle, WA), downstream of the human H1 promoter, resulting in IKK1-SuperA and IKK1-SuperB plasmids. A Control-Super plasmid, containing a scrambled nonspecific sequence that did not have relevant homology for IKK1 or other known murine sequences, was also constructed. The general structure for these oligonucleotides was BglII linker—sense sequence (ss)—hairpin spacer—antisense sequence (as)—end of transcription signal—HindIII linker (i.e., 5′-GATCCCC-ss-TTCAAGAGA-as-TTTTTGGAAA-3′). Sense sequences for each plasmid are shown in Figure 1(a).

### 2.2. Cell Culture and Treatments

HEK-293T cells were grown in DMEM (Gibco-BRL; Life Technologies) supplemented with 10% of FCS and antibiotics. Exponentially growing p60 culture plates were transfected by the calcium phosphate method with a total amount of 6 *μ*g of DNA containing both pRC-*β*actin-HA-IKK1 (a plasmid for the expression of a HA-tagged IKK1 protein [23]) and one of the pSuper-derivatives described previously at different ratios (Figure 1). As an additional control, we also performed cotransfections with an empty pSuper vector, unable to produce any shRNA. After 48 hours, cells were harvested and frozen for posterior analysis by northern or western blots.

### 2.3. Generation and Genotyping of IKK1-siRNA Transgenic Mice

**Figure 2:**
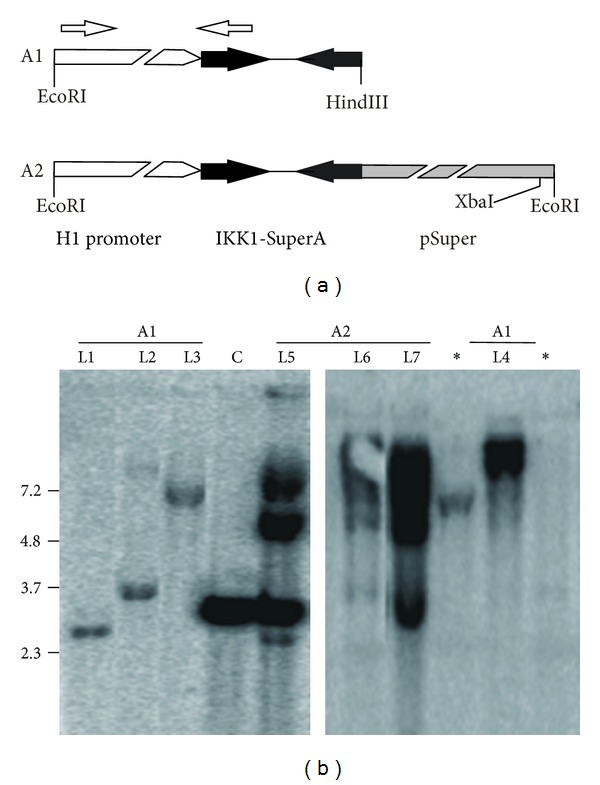
Generation of H1-IKK1-siRNA transgenic mice. (a) Schematic representation of the transgenes microinjected in one-cell mouse embryos. Transgene A1 is a 285 bp fragment containing the H1 promoter (white box) and the DNA oligonucleotide for shRNA synthesis (filled in black). Transgene A2 contained, in addition, the rest of the pSuper plasmid in its 3′ end (3234 bp total length). White arrows indicate the primers used for PCR-genotyping of transgenic mice. Relevant restriction sites are indicated. (b) Southern blot analysis of transgenic founders. The transgene injected (A1 or A2) and the name of the transgenic lines (L1 to L7) are indicated. Asterisks indicate founder mice that did not transmit the transgene to their progeny and were not included in further analysis. In the lane marked as C it was loaded DNA from a nontransgenic control mouse and the amount of fragment A2 corresponding to 20 copies per haploid genome. Figures in the left of the blot indicate fragment size in kbp.

Transgenic mice were generated by microinjection of the indicated constructs into (C57BL/6J × DBA/2J) F2 embryos using standard techniques [24] and transgenic lines were maintained by crosses with (C57BL/6J × DBA/2J) F1 mice. IKK1-siRNA transgenic mice were genotyped by southern blot or PCR analysis of tail genomic DNA. In southern blots, genomic DNAs were digested with XbaI (that cuts once in transgene A2) and the ^32^P-labelled fragment A1 was used as a probe (Figure 2(a)). For PCR amplification, we used primers for the amplification of a 221 bp-fragment from both transgenes (forward primer: 5′-GTCATCAACCCGCTCCAAGG-3′; reverse primer: 5′-GACTGACGGGGGATCTGTGG-3′). We used nontransgenic littermates as control animals. All experimental procedures were performed according to European and Spanish laws and regulations.

### 2.4. Protein and RNA Analysis

Whole-cell protein extracts from mouse organs and tissues were prepared as described in [25]. Protein content was determined by the Bradford colorimetric protein assay (BioRad Laboratories; Hercules, CA, USA). For the western blot shown in Figure 1(c), we used an antibody specific for HA epitope (Roche; Basel, Switzerland).

For northern blot assays, the organs studied were dissected from mice and immediately frozen in liquid nitrogen. Total RNAs from murine tissues were extracted with an acid guanidinium thiocyanate-phenol-chloroform mixture, as described in [26]. RNAs from cultured cells were extracted using TRIzol reagent (Invitrogen; Life Technologies Ltd. Paisley, UK) according to manufacturer's instructions. RNAs were quantified by 260 nm spectrophotometry and their quality was evaluated by electrophoresis.

To detect siRNA, 100 *μ*g of total RNA was loaded on 15% polyacrylamide −8 M urea gels (UreaGel System, National Diagnostics; Atlanta, USA) and separated by electrophoresis. Gels were transferred onto nylon membranes (Amersham Hybond-N+, GE Healthcare; Uppsala, Sweden) using wet transfer. We used as a probe a DNA oligonucleotide complementary to the IKK1 siRNA A (Figure 1(a)). These oligonucleotides were radiolabeled in their 5′-end by using polynucleotide kinase (Roche) and *γ* ^32^P-dATP. Band sizes were estimated by comparison with the bands of Decade Marker (Ambion; Austin, TX).

Equal loading was confirmed by using a U6 probe. We used Molecular Imager FX imaging system and the Quantity One software (BioRad Laboratories) for quantification of the intensity of the bands.

For the study of H1 RNA expression, a 345 bp fragment of the *RPPH1* gene was PCR amplified and used as a probe [27].

### 2.5. Histological Analysis of Tissues

Mouse tissues were dissected and immediately fixed in 10% buffered formalin or 70% ethanol and embedded in paraffin. Five *μ*m thick sections were used for H&E staining.

### 2.6. qRT-PCR Analysis

RNAs were purified with RNeasy Mini Kit (Qiagen; Hilden, Germany) and their integrity was analysed in a 2100 Bioanalyzer (Agilent; Santa Clara, CA, USA). cDNAs were obtained using a Transcriptor kit (Roche). For qRT-PCR, we used Taqman MGB probes with 6-FAM reporter dye (Applied Biosystems, Life Technologies Corporation; Carlsbad, CA, USA). We used probes specific for IKK1 (Mm00432529_m1) and for TBP (TATA binding protein, Mm00446937_m1). TBP values were used as a control for normalization.

We analysed samples in triplicate and at least two times in a Rotor-gene thermocycler (Qiagen; Germantown, MD). We quantified the IKK1 expression level in each sample as a percentage of the TBP-normalised average value obtained for IKK1 in control mice.

### 2.7. Mice Depilation

Mice were shaved with a clipper and then the stubble was removed using Veet depilatory cream (Slough, UK) following the instructions of the vendor.

## 3. Results

### 3.1. Selection of an Effective Sequence for Interfering IKK1 Expression

We used the software available at the Oligoengine web site (http://www.oligoengine.com/) for the selection of oligonucleotide sequences likely to knockdown IKK1 expression. We selected the sequences A and B shown in Figure 1(a) that did not show homology with other genes in the murine genome, thus lowering the chance of off-target effects. As a negative control, we used a sequence not complementary to IKK1 or to any other sequence found in the mouse genome (oligonucleotide C, originating Control-Super construct, Figure 1(a)). To evaluate the efficiency of these constructs for IKK1 knockdown, we cotransfected HEK293T cells with different ratios of plasmids directing the expression of an HA-tagged murine IKK1 protein and each of the shRNAs described in Figure 1(a). As an additional control we also transfected HEK293T cells with the HA-tagged IKK1 expression plasmid and an unmodified (empty) pSuper vector, unable to produce any shRNA. Analysis of IKK1 mRNA (Figure 1(b)) and protein (Figure 1(c)) expression 48 hours after-transfection showed that both IKK1-SuperA and IKK1-SuperB constructs were able to knock down the target gene at the mRNA and protein levels, being IKK1-SuperA more efficient. Furthermore, its efficiency was dose-dependent in the range tested.

### 3.2. Generation of Transgenic Mice

To assess whether H1-driven constructs are useful to knockdown gene expression in the skin and other organs of transgenic mice, we generated transgenic mice carrying the IKK1-SuperA construct, the most efficient design for IKK1 knockdown in our *in vitro* test. Since silencing by proximal prokaryotic sequences has not been studied for RNApol III-driven constructs, we microinjected both the whole linearized IKK1-SuperA plasmid including the prokaryotic sequences (a EcoRI-linearized 3234 bp-long construct, transgene A2, Figure 2(a)) and a smaller fragment containing only the H1 promoter and the DNA fragment coding for the shRNA (a 285 bp EcoRI-Hind III fragment, transgene A1, Figure 2(a)).

Microinjection of fragments A1 and A2 into the pronuclei of (C57BL/6J × DBA/2J) F2 embryos yielded 99 and 49 newborn mice, respectively. Out of them, six newborn mice that had been microinjected with transgene A1 and three with transgene A2 resulted positive in a PCR analysis for transgene presence (not shown). Transgenic status was further verified by southern blot analysis using as a probe the A1 fragment labelled with ^32^P (Figure 2(b)). Genomic DNAs were digested with XbaI, which cuts once into the A2 transgene and does not cut into the A1 transgene. Therefore, a 3.2 kbp band is expected in head-to-tail array integrations of the transgene A2 if the digestion is complete and the XbaI site located in the 3′-region of the transgene is conserved in all the integrated copies. The presence of bands of higher molecular weight suggests an incomplete digestion or the lack of the XbaI site in some of the copies. A1 founders would render a band of variable size, depending on the location of the XbaI sites surrounding the integrated transgene array. Differences in the intensity of the bands obtained in founder mice would reflect differences in the number of integrated copies of the transgene for each founder. Southern blot analysis indicated that A2 transgenic founder mice carried more copies than A1 founders. Except for the two mice marked with asterisks in Figure 2(b), all founders transmitted the transgene upon breeding, thus originating four transgenic lines for the A1 transgene (L1 to L4) and three for the A2 transgene (L5 to L7). Transgenic mice of all lines were viable and fertile and did not show any overt phenotype.

### 3.3. Expression of IKK1 siRNA in Transgenic Mice

**Figure 3:**
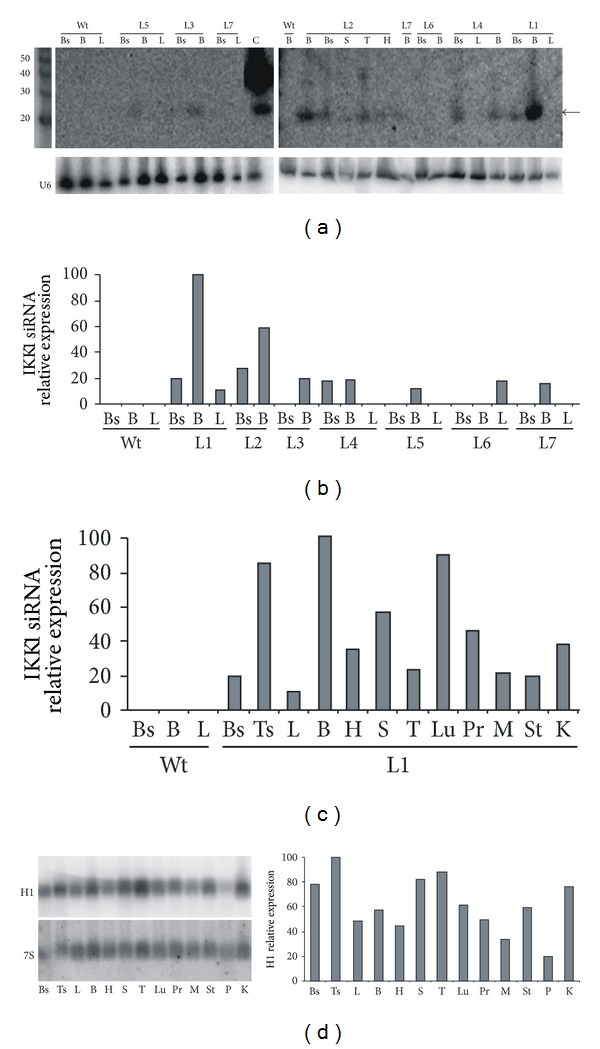
Expression of IKK1 siRNA in transgenic mice. (a) Upper part: representative examples of the northern blots performed for analysis of IKK1 siRNA expression in organs from H1-IKK*α*-siRNA transgenic lines. Lane C is a positive control that contains RNA from the transfected cells loaded in the first lane of Figure 1(b). The black arrow indicates the position of the specific 21-nucleotide band. Samples from wild-type animals gave no signals for IKK1 siRNA. The motilities of 20-, 30-, 40-, and 50-nucleotide RNA fragments are indicated. Lower part: the same blots were hybridized with a probe specific for U6 RNA, as a loading control. (b) Relative expression levels of IKK1 siRNA in different transgenic lines; siRNA intensities were normalized against their corresponding U6 signals and presented as a percentage of the value obtained for brain in line L1. (c) Relative expression level of IKK1 siRNA in organs of L1 transgenic mice. Signals obtained with an U6 probe were used for normalization. (d) H1 expression in control mice. The same blot was hybridized with a 7S-specific probe, as a loading control. In the histogram of the right, the relative expression of H1 normalized against 7S signals in different organs of control mice is shown. B: brain; Bs: back skin; H: heart; K: kidney; L: liver; Lu: lung; M: muscle; P: pancreas; Pr: prostate; S: spleen; St: stomach; T: thymus; Ts: tail skin.

Transgene expression was first analysed in back skin, brain, and liver of all transgenic lines by northern blot using a ^32^P-labelled oligonucleotide A probe and a U6 RNA (a ubiquitously expressed component of the spliceosome) probe for loading normalization. Representative results are shown in Figure 3(a), and a summary of the normalized signals obtained for the different lines in several northern blots is shown in Figure 3(b).

We found substantial differences in the expression level of the siRNA among the different transgenic lines, being L1 the line with the highest expression level. Lines bearing the transgene containing pSuper backbone sequences (lines L5 to L7) showed lower expression level than lines lacking these plasmid sequences (L1 to L4). These results indicate that, as described for transgenes transcribed by RNApol II [16], it is advisable to delete plasmid sequences in order to get high expression levels in transgenes containing the H1 promoter. In addition, the relative expression level of the transgene was not the same in the tissues studied in the different transgenic lines, as might be expected for transgenes driven by the promoter of H1 (a ubiquitously expressed small RNA gene). Expression in brain was higher than in back skin in some lines—five times in line L1 and two times in line L2—but both organs expressed the transgene at the same level in line L4 (Figure 3(b)). These results suggest that transgenic constructs containing the H1 promoter are sensitive to position effects, in a similar way as classical RNApol II-dependent transgenic constructs.

We selected the L1 line to analyse in more depth the expression pattern of the transgene. We extracted RNAs from 12 different organs and analysed by northern blot the amount of siRNA produced in hemizygous animals. As shown in Figure 3(c), the expression levels varied greatly in the different tissues tested, with differences of up to 10-fold. This could be due to differences in the transcription rate of H1 promoter in different organs, in accordance with the described cell type specificity of transcription for RNApol III-dependent genes [13]. Alternatively, the activity of the enzymatic machinery needed for the production of siRNA from the shRNA could be different in different organs. In order to test these possibilities, we studied the expression level of endogenous H1 gene in a similar set of organs and found marked variations between them (Figure 3(d)), although to a lesser extent than those found for the IKK1 siRNA transgene. In addition, the differences in H1 expression did not parallel those found for IKK1 siRNA. These data suggest that the differences in transgene expression could be caused, at least partially, by intrinsic differences in the rate of transcription of transgenic constructs containing H1 promoter in different cell types. However, as there is not an accurate correlation between the expression level of IKK1 siRNA and H1 in the organs tested, other factors might additionally be affecting transgene expression.

### 3.4. Reduced Expression of IKK1 in Transgenic Mice

**Figure 4:**
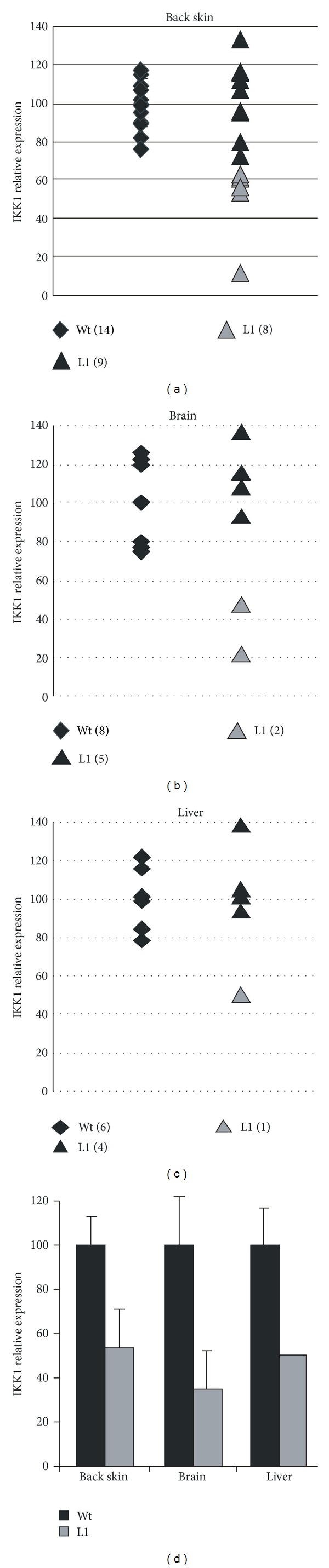
Reduced IKK1 mRNA expression in some H1-IKK1-siRNA transgenic mice. (a) IKK1 qRT-PCR analysis in back skin. Each value is presented as a percentage against the media obtained for wild-type mice. Diamonds indicate the values obtained for Wt mice and triangles those obtained for Tg mice. Grey triangles represent Tg mice with diminished (65% or below) IKK1 expression level. The number of mice analysed belonging to each category is indicated. ((b), (c)) Similar analysis as in (a), but for brain and liver, respectively. (d) Bar chart of the expression level of those L1 mice with diminished IKK1 expression level.

To assess the capability of the H1-driven transgene to knockdown IKK1, we studied IKK1 expression in some organs from L1 transgenic mice. We selected back skin (as IKK1 absence leads to profound phenotypic alterations in skin [17, 18]); brain, which is the organ with the highest level of IKK1 siRNA expression (Figure 3(c)); and liver, which expresses a remarkable high level of IKK1 mRNA (not shown). We performed qRT-PCR analysis of IKK1 expression in organs from several L1 transgenic mice and nontransgenic littermates. A summary of the results obtained is shown in Figures 4(a)–4(c). Of note, we found for the three organs tested greater variability of IKK1 mRNA expression levels in mice transgenic for the IKK1 siRNA construct than in nontransgenic littermates. Some transgenic mice expressed IKK1 roughly at the same level than Wt mice (black triangles in Figures 4(a)–4(c)) and other showed decreased expression in relation to Wt mice (less than 65% of the average expression in Wt mice; see grey triangles in Figure 4(a)). The proportion of transgenic mice with decreased IKK1 expression varied from 20% in liver to 47% in back skin. When considering only values for mice with decreased expression, IKK1 mean expression was between 35 and 55% of the values found in Wt mice, depending on the organ (Figure 4(d)). These results indicate a variable efficiency in IKK1 knockdown between different mice, as not all of them showed decreased IKK1 mRNA. It is interesting to note that there is no apparent correlation between the expression level of IKK1 siRNA and knockdown efficiency in the tested organs, as brain showed around 5-fold more IKK1 siRNA expression than back skin or liver (Figure 3(c)), but this difference of transgene expression did not correlate with IKK1 inhibition efficiency (Figure 4).

Immunohistochemical staining of paraffin sections of skin with an antibody specific for IKK1 revealed variations in the intensity of staining along the histological section both for Wt and Tg mice. Interestingly, under the same reaction conditions, some of the Tg mice (5 out of 11) showed weaker staining or more marked variations than Wt mice, probably reflecting a greater variability in IKK1 expression in skin keratinocytes of L1 transgenic mice (not shown) as a consequence of IKK1 inhibition in a fraction of the cells.

Taken together, our qRT-PCR and immunohistochemical analyses indicate that some L1 transgenic mice show a variable reduction in IKK1 expression in the different organs tested; this reduction does not seem to be uniform, as it did not affect every transgenic mouse and it did not affect every organ in a given mouse. In addition, even when considering an organ with clear IKK1 expression inhibition such as skin, not all the cells seem to be affected to the same extent.

### 3.5. Phenotypic Alterations in IKK1 siRNA Transgenic Mice

**Figure 5:**
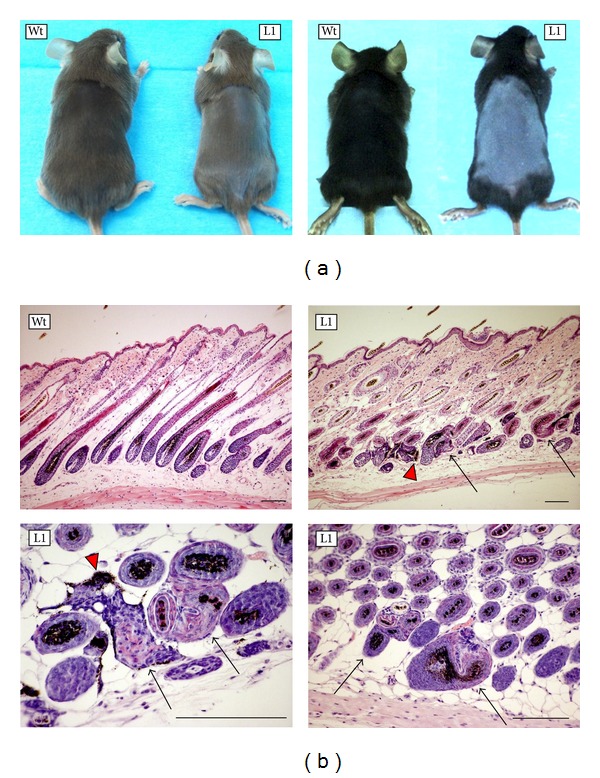
Most H1-IKK1-siRNA transgenic mice showed delayed hair follicle regrowth and hair follicle anomalies after depilation. (a) Slight (left) and severe (right) delay in the regrowth of hair follicles 11 days after depilation in transgenic mice. (b) Hematoxylin and eosin staining of skin histological sections. Morphological alterations in hair follicles of transgenic mice (black arrows) and dermal melanin deposition (red arrowheads) are shown. Bar: 80 *μ*m.

Considering the decreased amount of IKK1 in some transgenic mice and the profound phenotypic consequences caused by the absence of IKK1 in mice [17, 18], we studied if IKK1 siRNA transgenic mice presented any phenotypic alterations. Careful examination of litters and posterior genotyping revealed that around 50% of the transgenic mice presented some irregularity in the macroscopic appearance of hair and a lower density in pelage; these features made some transgenic mice distinguishable from wild-type littermates to an expert eye. This slight hair coat phenotype was observed in the first hair growth cycle but disappeared and was no longer detectable after weaning. In order to reveal hypothetical malfunction of hair follicles in siRNA IKK1 Tg mice, we subjected hair follicles to stress by removing the hair from back skin. We depilated 11 transgenic mice and 9 nontransgenic littermates at the age of 24 days, when hair follicles are at the end of the resting phase (telogen) of the first growth cycle. All the control mice showed a rapid hair regrowth, evident 4 days after depilation, when new hair began to cover the skin again; by 11 days after depilation, hair growth was complete, and the skin was totally covered with new hair (see examples in Figure 5(a)). By contrast, hair regrowth was delayed in 7 (64%) of the transgenic mice at 11 days after-depilation, ranging from an evident delay in growth (e.g., L1 transgenic mice in the left photograph of Figure 5(a)) to a complete lack of hair coat regrowth (see L1 mice in the right image of Figure 5(a)).

In the histological analysis of the back skin of these mice, we occasionally observed some distorted hair follicles in both Tg and Wt mice, probably because of the stress associated to depilation. The percentage of altered hair follicles was greater in Tg mice than in Wt littermates (on average, 6.3% in Tg versus 2.9% in Wt mice). These abnormal follicles were characterised by distorted shapes and follicle degeneration, accompanied by dermal deposition of melanin and local infiltration of mononuclear cells (Figure 5(b)) around the follicular debris. In addition, transgenic mice presented irregularities in the distribution of melanin in the medulla of the hair shaft and the presence of some morphologically distorted hair shafts, which did not correspond to any of the usual hair types found in control mice—guard, awl, auchene, and zig-zag hairs—(not shown).

In summary, siRNA IKK1 Tg mice showed more severe alterations in the shape of hair shafts, and in the growth and shape of hair follicles, than those found in wild-type littermates after depilation. It is interesting to note that we did not detect decreased levels of IKK1 mRNA in all the mice that showed phenotypic alterations. Considering the observed variability in IKK1 knockdown efficiency between different organs and mice, it could well be that knockdown efficiency varies also between neighbour cells within a given organ, as described by others [28]. This situation would result in sporadic cells or groups of cells with diminished IKK1 expression, so that they will cause local phenotypic alterations that could not be translated to a detectable decrease in IKK1 mRNA level in a tissue extract.

## 4. Discussion

In this report, we have studied transgenic mice expressing a siRNA specific for IKK1 under the transcriptional control of human H1 promoter. We generated these mice by microinjecting different fragments of a pSuper-derived construct containing the interference sequence into one-cell embryos. We studied transgene expression in brain, back skin, and liver of several lines and found that those lines that included the pSuper backbone in the injected fragment expressed poorly, and frequently only in a fraction of the tissues tested. By contrast, those lines lacking the plasmid backbone expressed the transgene at higher levels than lines carrying the plasmid backbone. Even more, some lines lacking the plasmid backbone expressed the transgene in all the tissues tested. Therefore, one of the conclusions drawn from this work is that it seems to be a good policy to delete prokaryotic plasmid sequences in transgenes transcribed by RNApol III, as it was also described for RNApol II-transcribed transgenes at the beginning of the transgenesis era [16].

We studied the expression pattern of the transgene in a broader set of tissues and organs from mice of L1 line, which showed the highest expression level in brain. In this line, every organ analysed expressed the transgene, although with marked differences in the level of expression. As these differences in expression could reflect intrinsic variations in the activity of H1 promoter in different cell types, we determined the abundance of H1 RNA in a similar set of samples. In accordance with the presumed ubiquitous expression pattern of H1 promoter, we found a detectable signal in all the organs tested. Of note, H1 expression level also varied among the organs, although with a different pattern than the siRNA IKK1 transgene. From all these data, we conclude that the transgene we used (and probably others containing the human H1 promoter or other RNApol III-transcribed promoters) is similar in several aspects to more classic RNApol II-transcribed transgenes. Firstly, our transgene is prone to position effects, displaying different expression patterns in different transgenic lines; secondly, its expression is not proportional to transgene copy number; and finally, it is sensitive to the presence of prokaryotic plasmid sequences. In order to overcome this position effect sensitivity and to get more reproducible expression patterns, it would be interesting to test the use of bigger and more active versions of the H1 promoter [29], or to include general or tissue-specific enhancers for increasing expression [30]. In addition, the use of insulators could also result in better expression of RNApol III-driven interfering constructs, as it was previously found for RNApol II constructs [31, 32].

siRNA IKK1 transgene results in a lower steady-state amount of IKK1 mRNA in different organs in some of the transgenic samples studied. Surprisingly, the average reduction in the expression level of IKK1 mRNA in those animals with reduced expression was roughly similar in all the tissues tested (brain, liver, and back skin, Figure 4(d)) even though the IKK1 siRNA was expressed at different levels in different tissues (see Figure 3(c)). It is also remarkable that IKK1 expression was variably decreased in each individual mouse. Thus, we found that, for back skin, the same transgene integration lowered IKK1 mRNA expression to less than 20% of the average level found in Wt mice in one mouse, to around 55–60% in seven mice of the same line, and did not decrease IKK1 mRNA at all in the other nine. The question why IKK1 expression is not reduced to a similar extent in every tissue of every transgenic mouse of the same line is an intriguing one. In this context, other interfering transgenes have been described which also show variable efficiency between different animals [33], between different tissues or cell types [34–36] or even in the same cell type, giving rise to a mosaic gene expression knockdown [28, 37]. Differences among tissues could have to do, at least partially, with differences in the ratio between siRNA and target mRNA or with intrinsic differences in the functioning of the interference machinery. Anyway, it seems that inhibition of a gene product by transgenic expression of a siRNA is subjected to more variability than classical overexpression of a protein of interest in transgenesis experiments. The differences we find between transgenic animals of the same line could also be due to genetic heterogeneity between mice, since our transgenic founders were F2 mice between C57BL/6J and DBA/2J strains and they were subsequently bred to F1 mice of that background, resulting in a mixed genotype different for each mouse. However, the fact that we did not find a consistent degree of inhibition in different organs of a given mouse is against this idea. Therefore, although variations in knockdown efficiency could have to do with genetic variability, there are probably additional players. Variability in knockdown efficiency seems to be a common phenomenon in interfering transgenic mice [28, 33–37], which could reflect some randomness in the silencing process. In this regard, it would be interesting to generate additional transgenic mice in order to determine if knockdown variability is diminished by improvements in transgenic design, by using pure inbred strains of mice or by increasing transgenic dosage (i.e., in homozygous animals or in compound hemizygous of several insertions).

It is worth noting that phenotypic alterations occurred in IKK1 siRNA mice when hair follicles were challenged by a depilatory treatment. The hair follicle phenotype we found has not been previously reported in the animal models hitherto published with altered expression of IKK1, that is, general and stratified epithelia-specific knockouts, as well as skin overexpression of IKK1 [17–20, 22]. These follicle alterations could be related to the multiple and complex functions of IKK1 in epidermal physiology. On the one hand, IKK1 gene inactivation leads to such profound alterations in embryonic skin that newborn mice die and the study of hair follicles in adult life is therefore precluded. In addition, the IKK1 protein has different targets and functions depending on its location in the cytoplasm or in the nucleus [38, 39]. Finally, as some of the functions of IKK1 are not cell-autonomous [40], dermal IKK1 can compensate for the lack of IKK1 in keratinocytes. Thus, the specific phenotype we have found may arise because of the simultaneous decrease, albeit at not well-defined rates, of IKK1 function in both dermal and epidermal cells. Interestingly, alterations in the shape of hair follicles are also found in mice expressing mutant forms of IKK1 in skin keratinocytes (our unpublished results). Therefore, the hair follicle phenotype described in this report is probably caused by changes in IKK1 activity, and not by other nonspecific effects of siRNA, as it has been described in other experiments involving RNA interference [41].

Taken together, the results described in this paper represent an example of the potential of the technology of RNA interference in transgenic mice. This kind of transgenic mice can be considered as complementary to other genetically modified models (mice with general and tissue-specific knockout—or overexpression—of the protein under study), and actually different models frequently render different valuable functional information. Our results also give interesting clues, to those aiming to knockdown a gene product using RNApol III-dependent transgenes, regarding the design of the transgene to be injected. In summary, we show that transgenic constructs directed by the H1 promoter can be used for the knockdown of genes of interest in different organs and for decreasing the level of the corresponding mRNAs in transgenic mice generated by pronuclear microinjection, and for the generation of relevant animal models complementary to knockout or overexpression transgenic mice in deciphering protein functions.

## Abbreviations

IKK: Kinase of the inhibitor of NF*κ*B
NF*κ*B: Nuclear factor *κ*B
RNAi: RNA interference
RNApol: RNA polymerase
shRNA: Small hairpin RNA
siRNA: Small interfering RNA
Tg: Transgenic
Wt: Wild type.
